# Suprachoroidal hemorrhage during phacoemulsification surgery associated with recent COVID‐19: A report of two cases

**DOI:** 10.1002/ccr3.6204

**Published:** 2022-08-09

**Authors:** Hossein Jamali, Elham Sadeghi, Mohammad Hossein Nowroozzadeh

**Affiliations:** ^1^ Department of Ophthalmology Shiraz University of Medical Sciences Shiraz Iran

**Keywords:** case report, cataract surgery, COVID‐19, phacoemulsification, suprachoroidal hemorrhage

## Abstract

To report two cases of suprachoroidal hemorrhage during otherwise uncomplicated phacoemulsification, in whom COVID‐19 was documented with a positive PCR test about 1 month before the surgery and the importance of postponing elective surgeries for several months after infection.

## Graphical abstract

COVID‐19 may involve vascular‐rich tissues such as the choroid, so it is best to postpone elective eye surgeries to prevent suprachoroidal hemorrhage.

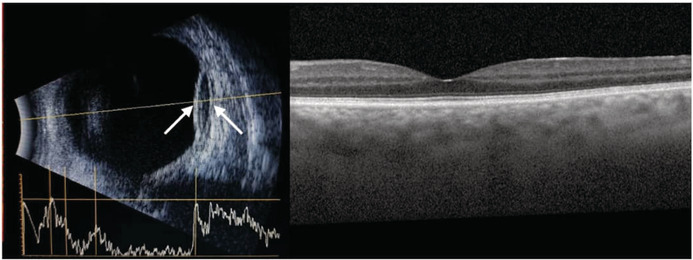

## 1 INTRODUCTION

Suprachoroidal hemorrhage (SCH) is a rare complication of intraocular surgery that could be devastating and associated with permanent visual loss in some cases.^1^ The reported incidence of SCH during cataract surgery is 0.03–0.13%, usually occurring in patients with known systemic and ocular risk factors.^2^ Angiotensin‐converting enzyme 2 is a receptor for SARS‐CoV‐2 and is highly expressed in ocular vascular endothelial cells. The choroid is the most densely vascularized tissue in the body.^3^

Herein, we present two cases with a recent history of COVID‐19 who developed SCH during uncomplicated phacoemulsification surgery.

## 2 CASE PRESENTATION

### 2.1 Case 1

The first case was a 60‐year‐old man who underwent planned phacoemulsification cataract surgery in the left eye. His medical history was unremarkable. The patient had a documented positive polymerase‐chain‐reaction (PCR) for COVID‐19 5 weeks before the operation when he developed fever, cough, and body pain for a couple of weeks. The baseline characteristics of the patient are summarized in Table 1.

**TABLE 1:** Characteristics of the two patients with recent COVID‐19 and suprachoroidal hemorrhage during planned phacoemulsification.

	Case 1	Case 2
Age, years	60	71
Gender	Male	Female
Systemic disease	None	Grade‐1 hypertension
Eye	Left	Right
BCVA	20/200	Light perception
IOP, mmHg	15	14
Slit‐lamp exam	3+ nuclear cataract	Mature cataract
Fundoscopy	Normal	Impossible^a^
Anterior chamber depth, mm	3.41	3.23
Axial length, mm	23.3	23.4
Intraoperative heart rate, (mean, maximum), bpm	(67, 75)	(122, 130)
Intraoperative systolic BP, (mean, maximum), mmHg	(75, 84)	(128, 140)

Abbreviations: BCVA, best‐corrected visual acuity; BP, blood pressure; bpm, beats per minute.

^a^ Preoperative B‐scan sonography showed attached retina and choroid with clear vitreous.

**FIGURE 1:**
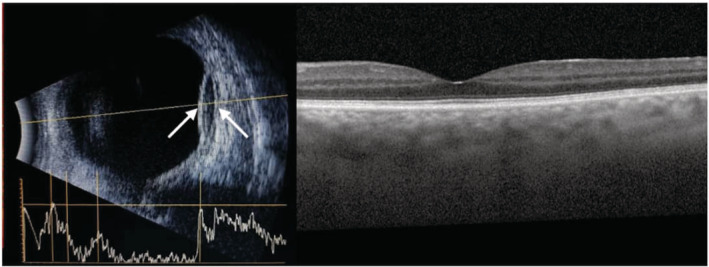
Left, the B‐scan ocular ultrasound image of Case 1 obtained 2 days after the surgery indicating the area of the suprachoroidal hemorrhage (between arrows). Right, EDI‐OCT image of the same patient took 3 weeks after the operation, showing normal macula with a subfoveal choroidal thickening.

The operation was uneventful till the irrigation/aspiration phase, when the anterior chamber suddenly became flattened, the posterior capsule attached to the cornea, and the iris prolapsed from the main incision. Intraoperative indirect ophthalmoscopy revealed temporal choroidal hemorrhage without the involvement of the posterior pole. The main corneal incision was sutured, and IOL insertion was postponed after the complete resorption of choroidal hemorrhage. The SCH was resolved within 4 weeks without any significant sequel (Figure 1). After the second surgery for IOL implantation, the final visual acuity returned to 20/20.

### 2.2 Case 2

The second case was a 71‐year‐old woman (Table 1) who underwent planned phacoemulsification surgery in her right eye under general anesthesia. She did not have any history of systemic diseases except for controlled stage‐1 hypertension. The patient had a history of COVID‐19 infection confirmed by positive PCR 4 weeks before the operation.

This patient also developed limited SCH in the inferior retina during the irrigation and aspiration phase. However, we could insert the IOL in the same theater. SCH was almost resorbed in 3 weeks. The final BCVA was 20/25.

None of the cases had a history of COVID‐19 vaccination, but their disease did not involve the lungs or required hospitalization, and they fully recovered within 2 weeks.

## 3 DISCUSSION

COVID‐19 exacerbation is partly attributed to a cytokine storm initiated by increasing levels of several pro‐inflammatory cytokines.^4^ Meanwhile, significant endothelial cell injury increases vascular permeability and leakage.^5^, ^6^ Although the lungs are the primary target for overwhelming inflammation and damage, several studies reported multiorgan failure due to systemic vascular involvement.^4^

The choroid is the most densely vascularized tissue in the body. Angiotensin‐converting enzyme 2 is a well‐documented receptor for SARS‐CoV‐2 and is highly expressed in ocular vascular endothelial cells.^3^ So, in principle, choroidal and retinal vessels could be affected by COVID‐19. In fact, recent studies reported microvascular and macrovascular retinal and choroidal changes in cases with COVID‐19.^3^, ^7^, ^8^ Although such involvements for choroidal vessels are not clinically supported so far, subclinical injuries from cytokine storm to these vessels are quite possible and might be a risk factor for SCH in patients undergoing intraocular surgery during the recovery phase of COVID‐19. This assumption is partly supported by the lack of known systemic or ocular risk factors of SCH in our patients and also their recent evident infection with COVID‐19. In the same setting, we had a rate of approximately 0.03% for intraoperative SCH during phacoemulsification in the pre‐COVID‐19 era (all in patients with known risk factors, systemic risk factors include old age and arteriosclerosis, coagulation disorders, vascular hypertension, diabetes mellitus, intraoperative tachycardia, and ocular risk factors include high myopia, glaucoma, previous vitrectomy, pseudophakia, and aphakia^9^), further increasing our suspicion of COVID‐19 as a predisposing factor for SCH in patients mentioned above.

Recent COVID‐19 infection might be a risk factor for SCH in patients undergoing cataract surgery. Although the two reported cases could not verify any cause‐and‐effect relationship, we should be vigilant about the possible association. Since COVID‐19 has the potential to make many known and unknown changes to the human body, it is wise to postpone elective surgeries for several months after infection to let the body completely recovers itself.

## AUTHOR CONTRIBUTIONS

Hossein Jamali and Elham Sadeghi contributed to study design and manuscript writing; Mohammad Hossein Nowroozzadeh contributed to writing/editing the manuscript

## CONFLICT OF INTEREST

The authors declared no potential conflicts of interest with respect to the research, authorship, and/or publication of this article.

## CONSENT

Written informed consent was obtained from the cases to publish this article and any accompanying image.

## Data Availability

It is on request from the authors. The data that support the findings of this study are available from the corresponding author upon reasonable request.
